# Dispersal Ecology Informs Design of Large-Scale Wildlife Corridors

**DOI:** 10.1371/journal.pone.0162989

**Published:** 2016-09-22

**Authors:** Robin A. Benz, Mark S. Boyce, Henrik Thurfjell, Dale G. Paton, Marco Musiani, Carsten F. Dormann, Simone Ciuti

**Affiliations:** 1 Department of Biometry and Environmental System Analysis, University of Freiburg, Freiburg, 79106, Germany; 2 Department of Biological Sciences, University of Alberta, Edmonton, T6G 2E9, Canada; 3 Faculty of Environmental Design, University of Calgary, Calgary, T2N 1N4, Canada; Universita degli Studi di Sassari, ITALY

## Abstract

Landscape connectivity describes how the movement of animals relates to landscape structure. The way in which movement among populations is affected by environmental conditions is important for predicting the effects of habitat fragmentation, and for defining conservation corridors. One approach has been to map resistance surfaces to characterize how environmental variables affect animal movement, and to use these surfaces to model connectivity. However, current connectivity modelling typically uses information on species location or habitat preference rather than movement, which unfortunately may not capture dispersal limitations. Here we emphasize the importance of implementing dispersal ecology into landscape connectivity, *i*.*e*., observing patterns of habitat selection by dispersers during different phases of new areas’ colonization to infer habitat connectivity. Disperser animals undertake a complex sequence of movements concatenated over time and strictly dependent on species ecology. Using satellite telemetry, we investigated the movement ecology of 54 young male elk *Cervus elaphus*, which commonly disperse, to design a corridor network across the Northern Rocky Mountains. Winter residency period is often followed by a spring-summer movement phase, when young elk migrate with mothers’ groups to summering areas, and by a further dispersal bout performed alone to a novel summer area. After another summer residency phase, dispersers usually undertake a final autumnal movement to reach novel wintering areas. We used resource selection functions to identify winter and summer habitats selected by elk during residency phases. We then extracted movements undertaken during spring to move from winter to summer areas, and during autumn to move from summer to winter areas, and modelled them using step selection functions. We built friction surfaces, merged the different movement phases, and eventually mapped least-cost corridors. We showed an application of this tool by creating a scenario with movement predicted as there were no roads, and mapping highways’ segments impeding elk connectivity.

## Introduction

The decline of biodiversity during recent decades has been largely attributed to habitat loss and fragmentation, as well as degradation of habitat quality [1, 2]. Intensity of resource extraction and road construction varies across the landscape due to land-use suitability and accessibility to humans [3]. This leads to fragmentation of the landscape into isolated habitat patches, surrounded by agricultural and altered forested landscapes [2, 4, 5]. As a result, conservation managers have started to identify regional conservation priorities and modify land management including the establishment of protected areas [6, 7].

Linear clearings, such as those created by power lines, pipelines, railways and roads, impact the environment with very different effects on species which can be either positive or negative [8, 9]. Linear features can facilitate alien plant invasions [10], negatively affect animal movement [8] causing reduction of home range habitat use due to avoidance of anthropogenic barriers [9, 11]. Movement patterns can also be affected in larger herbivores [12–15], resulting in a disruption of migratory behaviour [16]. Alternatively, high human activity on roads can displace predators but not prey species, creating spatial refuge from predation [17], interfering with predator-prey interactions via trait-mediated direct effects (i.e., human disturbance displacing large carnivore predators) and imposing indirect effects (e.g. human disturbance reducing predation risk for prey species, [17]).

Conservation efforts for large mammals by wildlife managers and conservationists typically focus on identifying and maintaining wildlife corridors to facilitate movement through human modified landscapes [3]. Poorly designed corridors can result in population sinks, wasted financial resources, or a loss of stakeholder support [18]. Recent and most-effective methods pursued are to model resistance surfaces derived from radiotelemetry data to determine least-cost-path (LCP) for best placement of corridors [19, 20]. However, using the proper tool for connectivity modelling may not be enough to achieve satisfactory results. Vasudev et al. [21] recently pointed out that connectivity modelling typically uses information on species location or habitat preference rather than movement, which unfortunately may not capture dispersal limitations or opportunities. Dispersal across landscapes, or the movement of individuals or genes among resource patches, is essential for functional connectivity. Vasudev et al. [21] recommended a change of focus for connectivity modelling from factors limiting dispersal to those that provide dispersal routes, and use these to identify geographic space where dispersal may be constrained.

**Fig 1:**
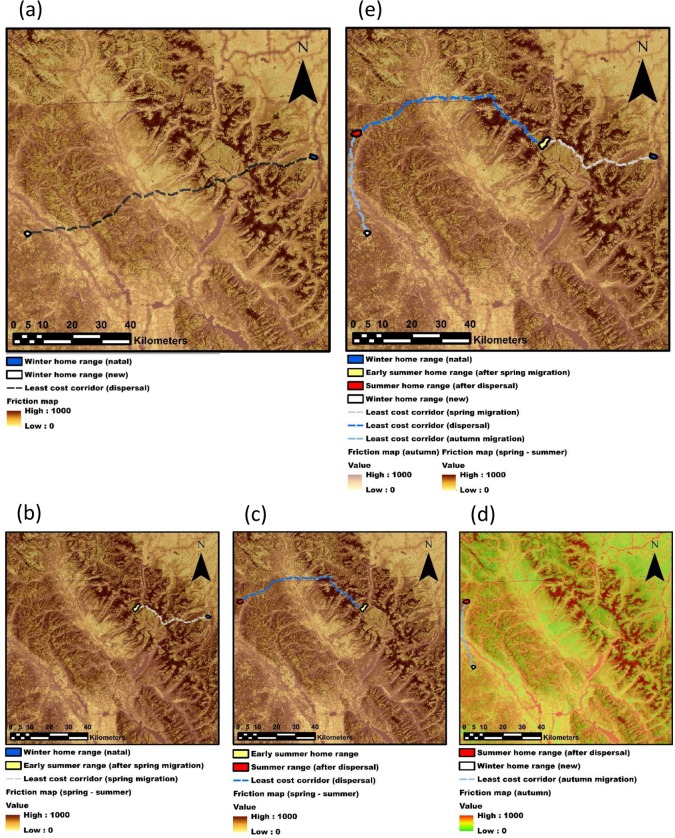
Conceptual figure illustrating the rationale behind the approach described in this study. The example refers to the identification of a wildlife corridor connecting two core areas, *i*.*e*., a hypothetical elk winter range and a new one reached after a dispersal event. Typical connectivity models (a) would depict the least cost corridor connecting the two areas, which is the most likely path given a friction map on the background. Our approach highlights the importance to implement behavioural ecology (in this case, dispersal ecology) into connectivity modelling science. A young male elk usually migrates during late spring—early summer with the mother’s group, moving from the natal winter range to the early summer range (b). During summer, the young elk may disperse to a new suitable summer home range (c), and, in autumn, eventually move to the new winter range (d). If the animal will adopt the migratory strategy, then it will periodically migrate between the new winter range and the new summer range (d). Implementing dispersal ecology into connectivity modelling means combining a sequence of migratory and dispersal movements (e, resulting from b + c + d), which suggests a very different potential wildlife corridor (e) compared to the one predicted by simply connecting the two winter ranges (a).

The goal of our study is to emphasize the importance of using a proper tool able to model connectivity with key information such as behavioural and dispersal ecology data of the target species. We built upon the findings of Squires et al. [22] and Killeen et al. [23] by integrating dispersal ecology with an applicable modelling tool. Squires et al. [22] combined broad-scale residency with fine-scale movement behaviour to depict linkages for Canadian lynx (*Lynx canadensis*). We adapted their method to examine movement ecology of young male ungulates, *Cervus elaphus*, in the Northern Rocky mountains. Killeen et al. [23] found that almost exclusively young male elk disperse and undertake exploratory movements, while females remain migratory or resident. Using these data provides an opportunity to analyse an unique dataset dealing with more than 50 young male elk monitored by means of satellite telemetry during a critical year of their life, i.e., when they undertake dispersal movements that are critical to gene flow. Our novel approach incorporates elk dispersal ecology into connectivity modelling science. Our method is based on the knowledge (e.g. [23]) that animals obviously do not move from wintering areas directly to new ones in one step (Fig 1A). Instead, elk move through the landscape and disperse to new areas through a sequence of concatenated steps undertaken from early spring, when young males leave the natal home range (*sensu* [23]), to late autumn, when they colonize the new winter range (Fig 1B–1E). Our paper is centred on habitat selection by young male elk during winter residency within the natal range, habitat selection during spring-summer movements–which is a mixture of migratory, exploratory, and dispersal bouts [23] (Fig 1)—habitat selection during summer residency, and, finally, habitat selection during autumn movements undertaken to colonize a new winter range. Our objective is to identify habitat drivers of landscape connectivity and thus infer how to construct wildlife corridors to promote gene flow at the landscape level, using unique movement data from animal dispersals, i.e. movement ecology of the age-sex class that drives population connectivity for this species [23]. Indeed, most research regarding corridor connectivity is limited by the availability of suitable data providing fine-scale information of habitat selection during the dispersal transience phase (but see [23]).

Our approach combined the use of resource selection functions (RSFs) [24], to identify and locate habitats selected during winter and summer residency periods [22], We used step selection functions (SSFs) to estimate habitat selection during spring and autumn movements undertaken by elk to connect winter with summer ranges and summer with winter ones, respectively. SSFs were used to calculate spring and autumn cost friction surfaces [25, 26] to predict least-cost corridors (LCC) connecting winter and summer residency areas [27]. First, we used these data to create a wildlife corridor network on a large scale following the recommendations by Sawyer et al. [28]. Second, we attempted to identify movement corridors that may have been lost due to the presence of major highways, thus highlighting highway sections impeding landscape permeability for potential dispersers.

## Materials and Methods

Our data collection complied with relevant federal laws of Canada and provincial laws of Alberta. Procedures were reviewed and approved by the University of Alberta Animal Care and Use Committee ACUC–Biosciences (Animal care protocol # 536–1003 AR University of Alberta, Edmonton, Canada), by all jurisdictions of the Alberta Government (Permit Numbers: BI-2008-19, RC-06SW-001 and 23181CN), and by Parks Canada (Permit Numbers: WL-2010-7292, WL-2010-5755).

Data handling and analyses were carried out using R [29]. Spatial analyses (*i*.*e*., mapping elk connectivity and defining wildlife corridors) were finalized in ArcGis [30].

### Study area

The study area covers a total of 46,000 km^2^ and is located in southwestern Alberta, Canada, and also adjacent portions of south-eastern British Columbia, Canada, and north-western Montana in the United States (S1 Fig). The landscape consists of the transition from flat agricultural grassland in the East to conifer and mixed hardwoods of the montane Rocky Mountain ecosystem in the West (elevation range: 900–3,400 m a.s.l.). There is considerable human activity in the study area dominated by cattle ranching and crop farming in the lowlands and forestry and gas natural extraction in the Rockies. The area has recreational hunting for elk from September to December [31]. The study area includes the Waterton Lakes National Park in Alberta and Glacier National Park in Montana (S1 Fig). Wolf (*Canis lupus*), cougar (*Puma concolor*), black bear (*Ursus americanus*), and grizzly bear (*U*. *arctos*) are the main elk natural predators in the region [17, 32].

### Elk captures and satellite telemetry data

In total, 182 elk were captured by this long-term monitoring program (http://montaneelk.com/) using helicopter net gunning, of which 62 were males (animal care protocol no. 536-1003AR University of Alberta). Captures occurred January through March in each year from 2007 to 2011. A vestibular canine was taken using dental lifters during the capture to assess age through cementum analysis (Matson’s Laboratory, MT, USA). All males were ˜1.5 years of age because net gunning was limited to capturing only young bulls with one-point (spike) antlers. Therefore, males were dispersing when just over 2 years old. Individuals were fitted with a radiotelemetry collar, programmed to a 2-hour relocation schedule. Data from radiocollars (Lotek ARGOS GPS Lotek Wireless Inc., Ontario, Canada) on males were received by email. Males made up a vast majority of the dispersing population, while most females remain resident or migratory [23]. Males were the focus of this study, whereas female data were screened to confirm migratory timing and define biologically meaningful seasons (see below). Of the 62 males captured, 54 individuals retained the collar or survived at least until September of each capture year. We restricted data to the first year after capture (146,233 GPS telemetry relocations referred to as ‘used’ relocations hereafter) when the majority of inexperienced young bulls explore new territories after leaving their mothers, and eventually disperse to new territories [23].

### Splitting the year into biologically meaningful periods

To model elk habitat selection during different phases of the colonization by young elk to new areas, as illustrated in our conceptual figure (Fig 1), we needed to define biologically meaningful periods when elk residency, migration, exploratory and dispersal behaviours are most likely to occur. To do so, we relied on the results of previous studies undertaken on the same elk telemetry database [23, 32, 33], which combined the timing of female elk migration behaviour [33] with male net displacement (ND *sensu* [34], shown in S2 Fig). Net displacement is a commonly used metric to distinguish movement patterns in telemetry data [34]. It is a time-dependent statistic used to measure straight-line distance between a starting location (i.e., capture site) and subsequent locations in a movement path of a given individual.

Male elk did not leave the winter or summer ranges thus we considered January to March ([23, 32], S2 Fig) winter residency and July/August to be a summer residency period ([23, 32], S2 Fig).

In our study area Killeen et al [23] found dispersal events ranged in length from 29 to 98 km (straight line distance from first to last location, actual distance travelled was greater). Duration of dispersal was 12 to 47 days (mean = 25.9 days), taking place between 18-May and 04-August, with the majority of movement occurring in June and early July. Exploratory movements, undertaken by all monitored males, were similar to dispersal ones in terms of timing and distance travelled but the animals returned to their starting location. We were keen to learn from both movement types because dispersal and exploratory movements are successful and unsuccessful dispersal events, respectively. Modelling habitat selection during these movement bouts allows us to understand both drivers and constraints of dispersal behaviour. Killeen et al [23] noted that exploratory movements were very similar to dispersal movements, and therefore individuals undertaking such exploratory loops were included as dispersers in their analyses, although they noted that results were not significantly altered by inclusion of these individuals. As a result, we considered the period from early April to late August to be spring/summer movements, referred to as spring movements hereafter. This time period includes the migration of young elk to early summer ranges with the mother’s group, as well as exploratory bouts and dispersal events. The use of broken-stick model (a threshold model, see below) allows the extraction of long movements from telemetry data collected from early April to late August, which represent data undertaken by animals to move away from the winter range and eventually disperse (Fig 1). Compared to short movements (e.g. resting, foraging), long movements are critical for building step selection functions and related friction maps (see below) used in connectivity modelling.

The majority of males did not migrate back to their previous winter range after their migration to summer range ([23], S2 Fig). After a successful dispersal, animals settled into a new home range and became either migrants or residents (Fig 1, [23]). Elk movements during autumn started as early as September and lasted to November. Autumn movements were very different from those in spring–early summer [23] and thus were modelled separately.

### Digital environmental data

A number of environmental covariates are known to influence elk movements in this region [23, 32]. These can be described using a combination of categorical land-cover maps and continuous measures of terrain, vegetation and distance to roads (S1 Table). Predictors for terrain features included a digital elevation model (DEM), from which we derived aspect, slope, and terrain ruggedness. The latter was calculated according to Riley et al. [35], and represents the topographic elevation difference from one cell on a grid to its eight neighbouring cells. We described land-use characteristics using a canopy cover model as well as seven categorical land-use types (S1 Table). The normalized difference vegetation index (NDVI) derived from 16-day MODIS satellite imagery was used as a proxy for forage quality [36]. Two seasonal NDVI layers (summer, winter) were generated during the months when elk showed habitat residency. Over the last decade, the NDVI has proven extremely useful in predicting herbivore and non-herbivore distribution, abundance and life history traits in space and time, and it has been established as a crucial tool for assessing vegetation phenology and primary productivity [37–41]. We created two road layers, containing the distance to double-lane highways, referred to as highways hereafter, and distance to smaller single-lane or gravel roads, referred to as gravel roads hereafter (Governments of AB & BC: National Topographic Database 1:50,000; U.S. Census Bureau Tiger/Line files, 2000). We focused on the use of broadly available data with consistent bias (resolution: 250 m) that were continuous across provincial borders between Alberta and British Columbia, and the international border with Montana. However, we used fine-scale environmental data when available for this large region (resolution: 30 m, S1 Table).

### Winter and summer habitat selection during residency period (Resource selection functions RSFs)

To identify winter and summer habitat ranges–defined as areas selected by elk during residency periods–we modelled population-level resource selection by elk using Resource Selection Functions (RSFs) following Manly et al.’s design II [24]. This sampling design implies the computation of resource selection by matching resources used at the individual level (i.e., satellite telemetry relocations) with resources available at the population level (i.e., random points drawn within the study area) [24]. Population-level RSF is the most suitable approach to identify residency core areas on a large spatial scale [22]. As a representative for available habitat, we randomly selected sample locations across the study area (defined by the 100% Minimum Convex Polygon MCP computed using all elk GPS relocations gathered by this long-term monitoring program). Each individual elk’s ‘used’ location was eventually associated to 10 random sample locations. Sensitivity tests performed using elk data and environmental predictors of this study site have shown that 10 random points per used location are sufficient to get stable parameter estimates in logistic regression models used to estimate resource selection functions (Ciuti S., unpublished).

Prior to development of the resource selection model, we screened environmental variables for collinearity using Pearson correlation coefficients (|*r*_*p*_| > 0:7) and used a variance inflation factor greater than 3 to drop multi-collinear variables [42]. Model coefficients were estimated using generalized linear mixed models (GLMM) with binomial distribution of errors, with individual elk as a random intercept [43]. We started with a full model, and variables that did not contribute towards the model based on Wald statistics (*p*>0.05) were not included. We included a quadratic term for all continuous predictors to allow for nonlinear relationships. For statistical modelling we used the R package *lme4* to estimates our GLMMs [44]. Using *β* coefficients estimated by the GLMM, we built a RSF of winter and summer according to Manly et al. [24], which takes the exponential form:

$$RSF\;scores=w(x)=exp(\beta_{1}{\text{x}}_{1}+\beta_{2}{\text{x}}_{2}+\ldots +\beta_{\text{i}}{\text{x}}_{\text{i}})$$ (Eq 1)

where *β*_*i*_ is the coefficient for each environmental predictor in a given resource unit *x*_*i*_ from a vector *x* of predictor covariates, and *w(x)* is proportional to the probability of selecting the resource unit [45]. The predictions of the RSFs were used to identify winter and summer core areas in a GIS framework [30].

The model’s performances were evaluated using the 5-fold cross validation approach introduced for RSFs by Boyce et al. [45], which involves calculating the correlation between RSF ranks and area-adjusted frequencies for a withheld sub-sample of data. We randomly divided telemetry locations in five subsets and then we withheld one subset for model prediction, while using the remaining four as model training data; we repeated this procedure for each of the subsets. The RSF scores gathered from each of the training datasets were placed into 10 bins [45]. Subsequently, we assessed each withheld subset against the predictions by the training model and compared them to each other using Spearman-rank correlation [45].

### Habitat selection during spring and autumn movements (Step selection functions SSFs)

Habitat selection during spring and autumn movements was computed by fitting step selection functions (SSFs, see [26] for a review). SSFs compare environmental attributes of observed steps (the linear segment between two consecutive observations of position of a tracked animal) with alternative random steps taken from the same starting point [26]. The straight-line distance (step length) between two successive locations was calculated using GME and R [29, 46].

Our goal was to understand habitat selection by elk as they undertake long movements (e.g., migratory, exploratory, and dispersal bouts), which is critical data required to identify wildlife corridors. To distinguish between long distance movements from shorter ones associated to foraging and resting, we fitted a broken-stick model in R with the package *segmented* [47, 48]. The broken-stick curve-fitting procedure defines a threshold, which has been shown to separate foraging vs. dispersal movements in large herbivores [47, 49]. This modelling approach characterizes an underlying nonlinear process that can give clues to behavioural mechanisms [49]. In practice, the broken-stick curve-fitting procedure calculates a threshold based on the log_e_ frequency distribution of movement rates for a given animal. Movement rates less that the threshold represent foraging movements, whereas movement rates greater that the threshold represent dispersal and other long movements [47]. In ecology, broken-stick models (a.k.a. change-point models) have a long history [50] and have seen application in a range of fields (see [51] and references therein; in movement ecology, see [23, 26, 47]).

To apply the broken-stick model to our study case, an equal number of locations was randomly selected for each animal to represent equal weightings and avoid biased estimates. For spring movements, this included all 54 elk with 800 randomly drawn steps per individual. Due to the shorter movement period in autumn and lower samples size from hunting mortality, each elk contributed 600 steps to the broken-stick model. All locations of spring and autumn movements with a greater movement speed than the observed broken-stick break point were used to build the SSFs.

Each long distance movement identified by the broken stick model was matched with ten random steps that we assumed to be available at each relocation [26, 52]. We reclassified the step lengths and turning angles of the long directional steps into bins of 50 m and 10 respectively, and sampled ten random steps from each bin in these distributions. We screened environmental variables for collinearity following the same procedure described for RSFs. Predictors included in the model were terrain ruggedness, canopy cover, distance to highways and to gravel roads, and land use (categorical variable). We included a quadratic term for all continuous predictors to allow for nonlinear relationships. Only roads in close proximity and with a traffic volume of at least 12 vehicles per day influence elk movements and behaviour in this region [31]. Therefore, we kept road distance layers at level when distance was farther than 2 km to highways (high vehicle traffic, [31]) and 1 km to gravel roads (low-medium vehicle traffic, [31]), so that step-selection functions in remote areas were not forced to estimate road selection where it does not affect elk [26].

Model coefficients were estimated using a mixed conditional logistic regression model [25, 53], where individual steps with their ten associated random steps were treated independently as strata and individual elk set as a random intercept (*i*.*e*. a mixed-effect model). Elk movements were modelled with the R package *mclogit* [54]. The step-selection function (SSF) assumes the same exponential form as in Eq 1. We built one SSF model for spring and one for autumn. Model robustness was evaluated using k-fold cross-validation for case-control design according to Fortin et al. [53], which differs to the k-fold cross validation used for validating RSFs. A SSF was built using 80% of randomly selected strata. This SSF was then used to predict the SSF scores for the remaining 20% of strata. The observed location of each stratum was ranked against its associated random locations and tallied. The bins’ ranking and associated frequency was carried out with Spearman-rank correlations (${\bar{r}}_{s}$). This procedure was repeated 100 times with replacement and the mean and 95% CI of ${\bar{r}}_{s}$ are presented for each model. The mean (${\bar{r}}_{s}$) and 95% CI were also presented under assumption of complete random patterns of habitat selection by following the same steps except that random steps were ranked against withheld random steps.

We mapped the SSF predictions spatially in ArcGIS and generated a relative probability surface. To use the SSF map as a source of cost friction, we inverted the map by subtracting the relative probability surface from one. In this study, the cell reflects resistance to habitat selection by young male elk moving through the yet unknown environment. Thus, we obtained two friction maps, one for spring movements, and one for autumn movements.

### Habitat selection during spring and autumn movements as if there were no roads

The two friction maps introduced above (spring, autumn) reflect the resource selection by elk as a function of all environmental predictors including roads. Using the same conditional logistic regression model structures fitted to obtain *β* coefficients for spring and autumn SSFs, we can again plug these *β* coefficients into the exponential form of the step selection function, this time setting the distance from roads to their maximum value. In practice, every pixel of the landscape would get high or low SSF scores depending on habitat suitability or unsuitability, respectively, no matter what the distance to the closest road is. We thus obtained two new friction maps (spring, autumn), this time produced as if there were no roads, which represent a scenario where elk would move through if there were no disturbance associated with roads [21, 55, 56]. Simply removing the distance to roads from the model relative to setting this explanatory variable to the maximum value would lead to model misspecification and biased estimates [57, 58].

### Mapping elk connectivity and defining wildlife corridors

To connect seasonally selected elk habitats, we connected true winter source locations with potential summer and future winter core areas identified by RSFs. It is important to clarify that we also allowed the movement of animals in the opposite direction, thus assuming that males can disperse into our study site. Firstly, using the isopleth tool in GME [46], we created 6 winter source core polygons from 50% contour of male locations during winter, grouped by herd. These corresponded to the six main locations (corresponding to six different wintering range) where monitored males were captured. Secondly, we depicted winter and summer core areas across the study area: based on the predictions of winter and summer RSFs, these core areas were those characterized by high RSF scores (i.e., RSF scores ≥ upper quartile), resulting in 11 winter and 15 summer core areas. We used the Linkage Mapper tool kit [27] to identify least-cost-corridors (LCCs) between core area pairs. We used the spring friction map, when predicting movement corridors connecting winter with summer areas, and the use of the autumn friction map, when predicting corridors connecting summer with winter areas. We calculated corridors connecting core areas that had a maximum Euclidean distance of 80 km (i.e., longest dispersal event ever recorded by a male prior to stop again in a stopover site). Linkage Mapper sums the cost-weighted distance (CWD) rasters from each core area pair, and normalizes least-cost-corridors (NLCCs) by subtracting the least-cost path distance (LCD) from the raw corridor (Eq 2, [27]).

$${\text{NLCC}}_{\text{AB}}={\text{CWD}}_{\text{A}}+{\text{CWD}}_{\text{B}}-{\text{LCD}}_{\text{AB}}$$ (Eq 2)

Linkage Mapper combines all NLCCs into one corridor map, using the mosaic function of ArcGIS [30]. The final corridor layer contains in each cell the minimum value of all NLCCs. Because Linkage Mapper produces a continuous output with CWD values, we arbitrarily cropped at a maximum CWD of 200,000 to improve the visualization of movement corridors. This is a user decision for visualization purposes and does not affect the movement corridor patterns. We carried out this procedure using spring and autumn friction maps, and repeated all the sequence using spring and autumn friction maps built as if there were no roads.

### Effect of roads on elk connectivity and wildlife corridor network

We estimated the highway length crossed by elk corridors in spring and autumn using 20 road segments from the highway network within the study site. We tested for the differences in the highway length (km) crossed by corridors between the two scenarios (i.e., including and not including the effect of roads) for spring and autumn movement using a paired t-test.

## Results

### Winter and summer resource selection predicted by RSFs

**Fig 2:**
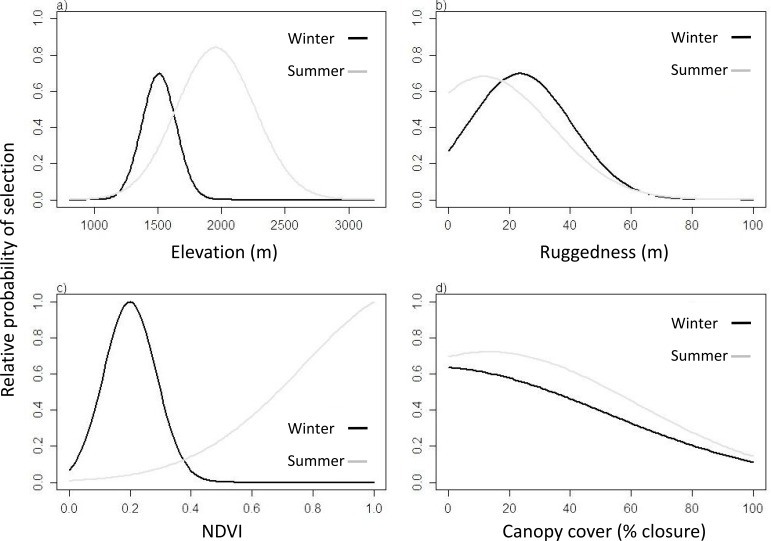
Relative probability of selection estimated by Resource Selection Functions (RSFs) in winter and summer for a) elevation, b) terrain ruggedness, c) normalized difference vegetation index NDVI and d) canopy cover.

All initial variables included in the full GLMM were retained with the exception of aspect (see S2 Table for full parameter estimates). Parameters estimated by the GLMM were used in the resource selection function, which we assumed to take the exponential form; resulting resource selection patterns for continuous environmental predictors were reported in Fig 2. RSF scores in presence-availability studies (i.e. w(x) values, see Eq 1) do not represent true probabilities [24]; they rather are proportional to the probability of selection [24]. RSF scores usually take the value w(x) ≥ 0. In order to improve figure readability, RSF scores were divided by their maximum value and thus rescaled within the interval 0 ≤ w(x) ≤ 1 (Fig 2). The higher the relative probability of selection obtained for a given predictor (with 1 as maximum possible value), the stronger is its role in driving selection (e.g., in Fig 2, NDVI is a stronger driver of resource selection by elk than canopy cover).

Elk selected for rugged open terrain at elevations around 1,500 m during winter, and flatter open terrain at higher elevations (approx. 2,000 m) during summer (Fig 2). NDVI was the main driver for selection in both winter and summer, followed by elevation, ruggedness and canopy cover (Fig 2). During winter, elk significantly selected grasslands, croplands, deciduous forests, and shrub lands over conifer forest (i.e., the reference category), whereas this was not true for mixed forest and the other land cover types (S2 Table). During summer, elk significantly selected deciduous forests, mixed forests, shrub lands, and grasslands over conifer forest (i.e., the reference category), whereas croplands were significantly avoided (S2 Table).

The Spearman rank 5-fold cross-validation suggests a very good predictive fit for each fold of the data, with *ρ* = 1 for each fold of summer, and *ρ* = 0.997, 1, 0.997, 0.997 and 0.997 for the 5 folds in winter, respectively.

### Elk resource selection predicted by SSFs during spring and autumn movements

The broken-stick model identified threshold speed during spring (6.97 m min^-1^) slightly faster than in autumn (5.87 m min^-1^, S3 Fig). Movement steps faster than the identified thresholds showed a forward directional tendency (S4 Fig) and were used to build our SSF models.

**Fig 3:**
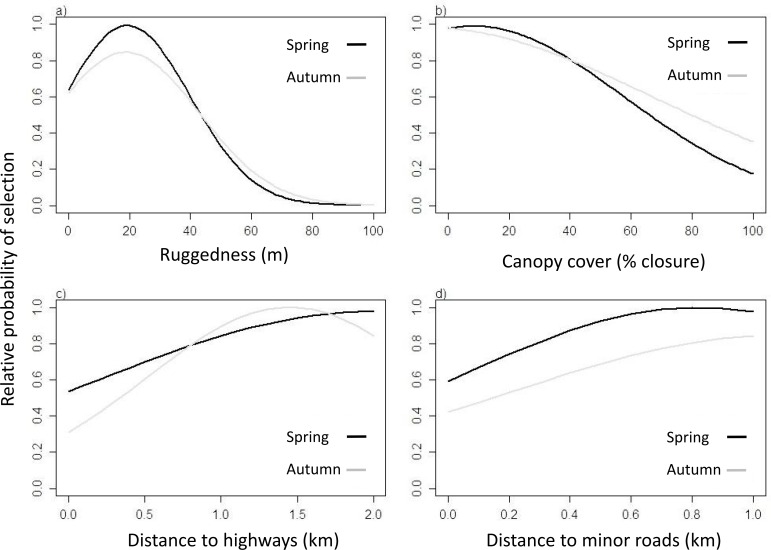
Relative probability of selection estimated by Step Selection Functions (SSFs) during spring and autumn movements for a) terrain ruggedness, b) canopy cover, c) distance to highways and d) distance to minor roads.

**Fig 4:**
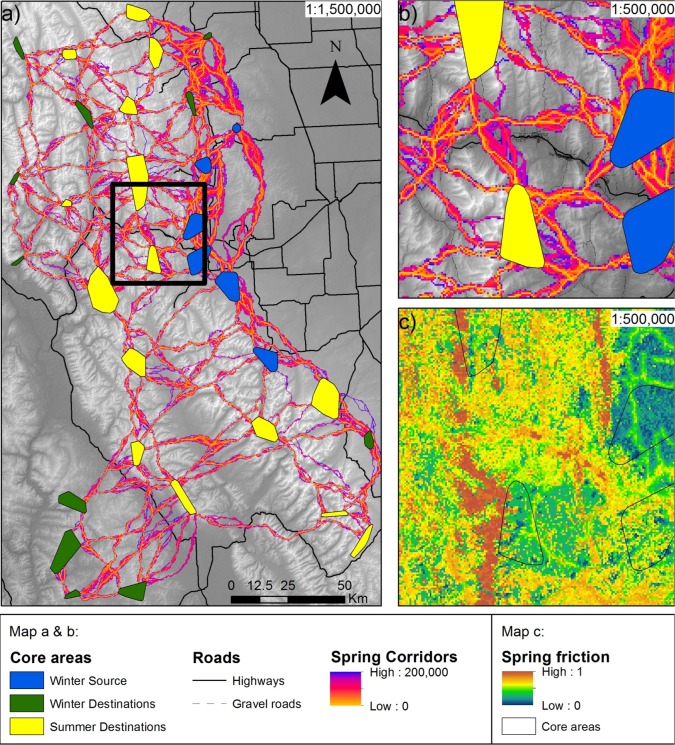
Corridor map of spring movements connecting winter ranges with summer ones. a) Normalized least-cost-corridors, with low values as optimal, connecting winter with summer core areas; b) close up section along highway 3; c) close up friction map produced from spring SSFs. Note that elk are predicted to move parallel or perpendicular to highway 3 (map b), in contrast to what is depicted in S5 Fig, where elk are predicted to move along highway 3 at the bottom of the valley, which would be the movement if there were no highway 3.

**Fig 5:**
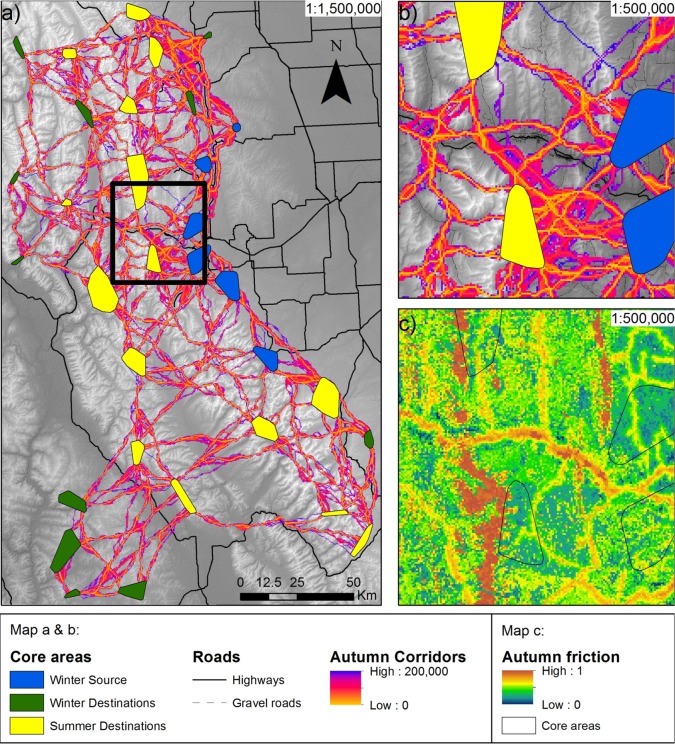
Corridor map of autumn movements connecting summer ranges with winter ones. a) Normalized least-cost-corridors, with low values as optimal, connecting summer with winter core areas; b) close up section along highway 3; c) close up friction map produced from autumn SSFs. Note that elk are predicted to move parallel or perpendicular to highway 3 (map b), in contrast to what depicted in S6 Fig, where elk are predicted to move along highway 3 at the bottom of the valley, which would be the movement if there were no highway 3.

During spring, elk significantly preferred to move through deciduous forests and grasslands (S3 Table), through slightly rugged terrain with little canopy cover, i.e., roughly 15% closure (Fig 3). In contrast, during autumn, elk did not significantly prefer to move through certain land cover types over others, but significantly avoided the land type ‘other’ which included urban areas (S3 Table). Compared to spring, elk preferred to move through slightly more rugged and less open terrain in autumn (Fig 3). Close proximity to roads was strongly avoided in both seasons. Predictions of SSFs were used to generate friction maps in a GIS framework that have been reported in Figs 4C and 5C. Predictions of SSFs were also used to generate friction maps as if there were no roads (S5 and S6 Figs).

The k-fold cross-validation suggested very good predictions from our SSF models (S4 Table).

### Elk habitat connectivity

Linkage Mapper produces a raster with cost distance values reflecting how costly it is to move between core areas. The values range from zero, where least-cost-path are optimal, upwards, reflecting higher cost for movement. On final corridor maps of spring and autumn movements, a cutoff value for CWD of 200,000 was used for visualisation purposes (Figs 4 and 5; see S5 and S6 Figs for corridors depicted under the scenario as if there were no roads).

### Effect of highways on elk movements and connectivity

**Fig 6:**
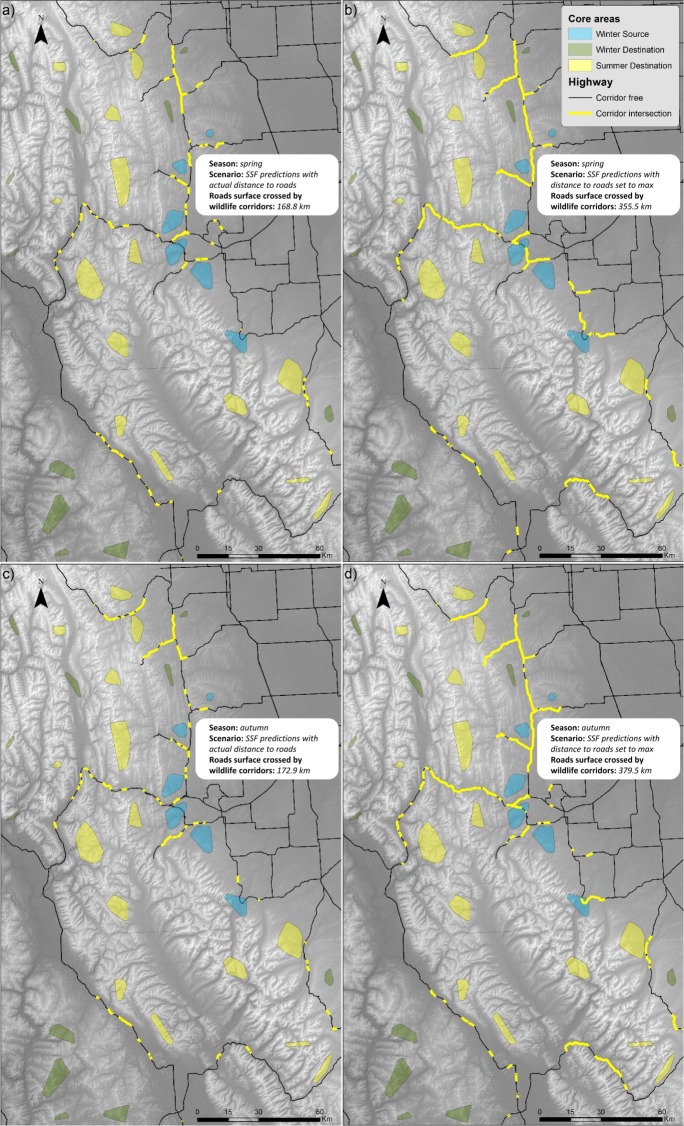
Corridor-road intersections of a) 168.8 km in spring considering SSFs predictions computed with actual distance to roads, b) 355.5 km in spring assuming as there were no roads (i.e., distance to roads set to maximum value when predicting SSFs), c) 172.9 km in autumn considering roads, and d) 379.5 km in autumn assuming as there were no roads. Maps b) and d) depict road segments that would be crossed by elk if there were no roads. About half of these segments are predicted not to be crossed by elk (maps a, c) as a result of road avoidance by elk.

Total corridor-highway intersection lengths were 355.5 and 379.5 km for spring and autumn, respectively, when we considered movement corridor network as if there were no roads (e.g., distance to road set to maximum value; Fig 6B and 6D). However, when actual distance to roads was used for predictions, total measured corridor-highway intersection lengths were reduced to 168.8 km for spring movements, and 172.9 km for autumn respectively (Fig 6A and 6C). After taking into account of the effect of roads on connectivity, we found a significant loss of corridor-highway permeability during both spring (paired t-test: in spring, *t* = -3.291, df = 19, *p* = 0.004) and autumn movements (*t* = -5.366, df = 19, *p*<0.0001).

## Discussion

Our novel approach takes into account the behavioural ecology of the target species when modelling wildlife corridors. We implemented dispersal ecology into landscape connectivity (*sensu* Fig 1), by using information about localities that the elk dispersed to and colonized based on a sequence of movements that were concatenated and spread over several weeks. Varying seasonal environmental conditions, such as bare deciduous forests in spring or after leaf fall as well as hunting pressure in autumn alter the movements of elk depending on time of the year [32, 59, 60]. We built SSFs taking into considerations these key periods of elk ecology. More importantly, spring friction maps were built to identify corridors selected by elk to move from winter ranges to summer ranges, when young elk initially migrate with mothers’ groups, undertake multiple exploratory bouts, and eventually disperse to new summer areas. Autumn friction maps were deployed to depict the corridors used to move from summer core areas to new winter areas potentially targeted by dispersers, which are very different movement types compared with those recorded in spring [23]. Indeed, once elk have found a new summer range, they likely meet other elk and their selection of movement habitats or routes would be with elk they have developed an association with, thus movements during autumn could reflect learned migration from other elk (*e*.*g*., Fig 1D).

We depicted summer and winter core areas based on RSF scores and connected them using the Linkage Mapper tool [61], following the rules of seasonal friction maps defined by SSFs. We showed how elk would move in absence of roads, and then imposed restrictions by roads. Subsequently, we quantified the loss of connectivity across the study area due to major highways. This provides insight on facilitating movement as well as identifying areas where it is impeded [61].

We focused on large-scale movements by young male elk. Wildlife corridors for any large mammal can be designed for different purposes, depending on specific movement types, such as small-scale movements undertaken to move between different foraging sites, or large scale migration [62]. Depicting large-scale wildlife corridors to improve gene-flow across a landscape ultimately requires data of the movements of those individuals potentially dispersing. Research has been limited due to the difficulty of maintaining an adequate sample size of dispersers, especially in hunted populations [63]. Dispersal by elk is dominated by males because young males leave their mother during their second-year spring migration, after snow has melted, when they then explore new and unknown landscapes [23]. Focusing on large-scale movement behaviour of all young males has been suggested to best reflect dispersal behaviour relevant to gene flow [64].

The wildlife-corridor network provided in this study differs from most other output from least-cost-path (LCP) analysis [22, 56]. In previous studies, source and destination locations are often placed at regular intervals or along a border rather than inside highly suitable residency areas across the landscape. Especially over large distances, these LCPs may run together into a single main corridor as seen by Cushman et al. [56] and Squires et al. [22]. However, young and inexperienced male elk do not follow a single most-suitable route during dispersal. There are many extrinsic factors, such as competition, high population density, or predator or human encounters for which we have not accounted. Such factors could potentially influence movement or influence travel direction [25]. Therefore, it is important to map multiple potential core areas across the landscape that act as nodes, and to create a network of corridors between them (Figs 4 and 5). Furthermore, the common ‘Cost distance’ and ‘Cost path’ tools of ArcGIS provide a corridor output of one grid cell width, which is not biologically meaningful [28, 30]. Linkage Mapper on the other hand, allows to produce a continuous output with CWD from the optimal LCP between core area pairs. This output is ecologically more meaningful compared with buffered single-line corridors [27].

### Application and future directions

Roads may strongly influence biological patterns, diversity and the integrity of wildlife communities [9]. Whittington et al. [65], for instance, highlighted how high road density has a strong effect on movement of wolves. Ciuti et al. [31] found that ungulates were negatively affected by roads (e.g. increased vigilance, decreased foraging) with increasing human activity (high vehicle traffic combined with recreational and hunting activities). Seidler et al. [16] also identified that major highways induce increased stopover in movement or provide complete barriers to migration of pronghorn (*Antilocapra americana*). Therefore, we created two scenarios with and without roads as part of the friction across the landscape that reflect movement separately for spring and autumn, highlighting highway sections potentially interfering with dispersal by young male elk moving between core areas. The scenario modelled with no roads, highlights highway sections where elk would have dispersed if a highway did not exist. The second scenario, where effect of roads is included, highlights highway sections where young elk most likely would cross to reach other core areas. Therefore, these sections would warrant the greatest conservation attention, e.g., highway crossing structures or warning signs to motorists.

We believe that the combination of advanced connectivity modelling tools with behaviour and ecology of target species could aid conservation managers as a tool to make better-informed management decisions, which in our study is to improve gene flow of elk across the landscape in the northern Rocky Mountains. The corridor map provided is not a single end-stage result. The friction maps can be used to investigate the connectivity and loss to roads by linking any areas of interest, such as protected areas, or to investigate the best place to locate a protected area to enhance connectivity. Furthermore, this information can be used in road development. Before placement of new roads, the influence of these on current movement corridors can be investigated and further they could be integrated into road-placement planning prior to construction.

Better understanding the dispersal and stopover ecology of any species is essential to adapt landscape resistance values and design functional wildlife corridors. Focusing on young males, the dispersing group in most mammals, is most likely to enhance gene flow [63, 66]. Applications are not restricted to migratory ungulates, and we could envisage applications for many other geographically restricted populations in other regions such as Africa or Asia [67, 68].

A next step in this study could be to use our spatio-temporal movement model of elk dispersal and compare it with the genetic differentiation of elk across the landscape. The potential link to genetics is quite an exciting prospect because finding a link between the structure anticipated by the dispersal model and the genetic structure of the population could explain a large amount of genetic variability across space [69]. In this way it would be possible to understand the actual effect of road barriers on the genetic diversity and population structure in the study area [70].

## Supporting Information

S1 Fig Study area map. (DOCX)

S2 Fig First year net displacement (ND, in km) in young male elk. (DOCX)

S3 Fig Broken stick model for spring and autumn elk movement rates. (DOCX)

S4 Fig Step lengths and turning angles of elk movements. (DOCX)

S5 Fig Corridor map of spring movements assuming there were no roads. (DOCX)

S6 Fig Corridor map of autumn movements assuming there were no roads. (DOCX)

S1 Table Digital data layers. (DOCX)

S2 Table Resource selection functions (RSFs) during winter and summer. (DOCX)

S3 Table Step selection functions (SSFs) during spring and autumn movements. (DOCX)

S4 Table K-fold cross-validation of SSFs. (DOCX)
