# Supplementary material for: Dispersal Ecology Informs Design of Large-Scale Wildlife Corridors
Source: PLoS One. 2016 Sep 22;11(9):e0162989. doi: 10.1371/journal.pone.0162989 (PMC5033395; doi:10.1371/journal.pone.0162989)
Supplement: S1 Fig — (DOCX) [file pone.0162989.s001.docx]

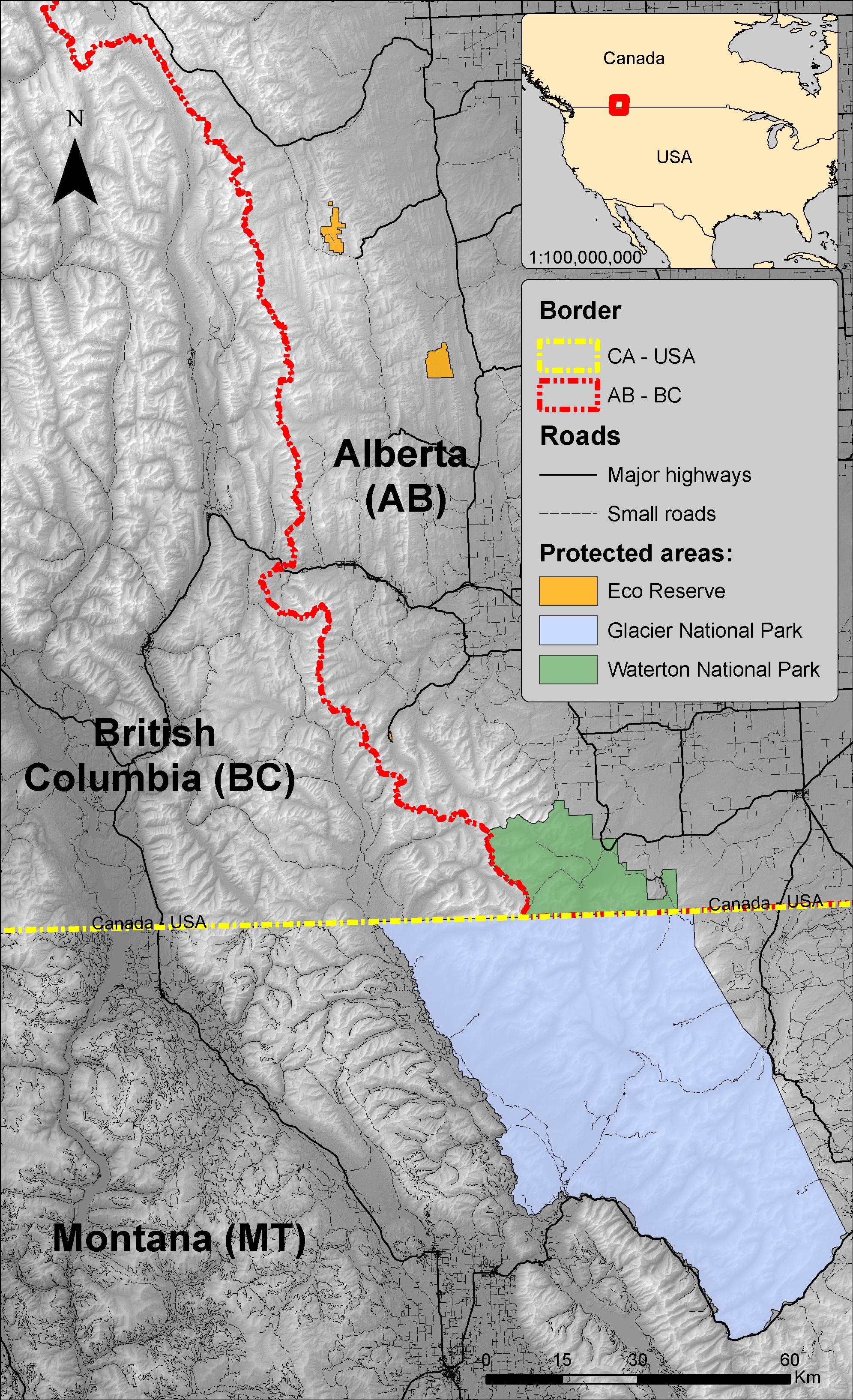


**S1 Fig -** Study area encompassing the provinces of Alberta (Canada) and British Columbia (Canada) and the state of Montana (U.S.A.).
