# Supplementary material for: Dispersal Ecology Informs Design of Large-Scale Wildlife Corridors
Source: PLoS One. 2016 Sep 22;11(9):e0162989. doi: 10.1371/journal.pone.0162989 (PMC5033395; doi:10.1371/journal.pone.0162989)
Supplement: S2 Fig — (DOCX) [file pone.0162989.s002.docx]

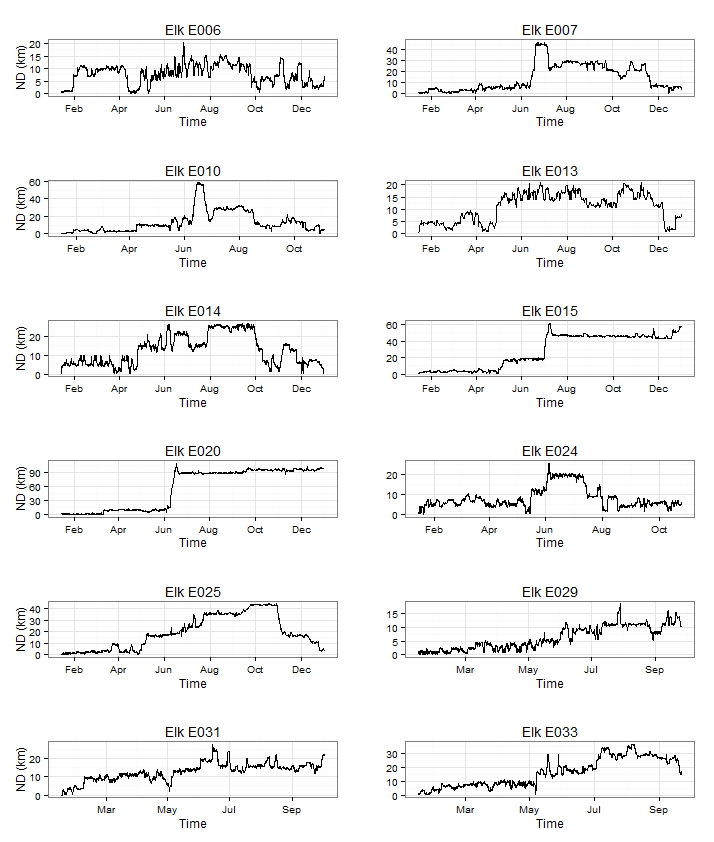


**S2 Fig -** Net displacement. First year net displacement (ND, in km) in young male elk (note the different scale on the y-axis).


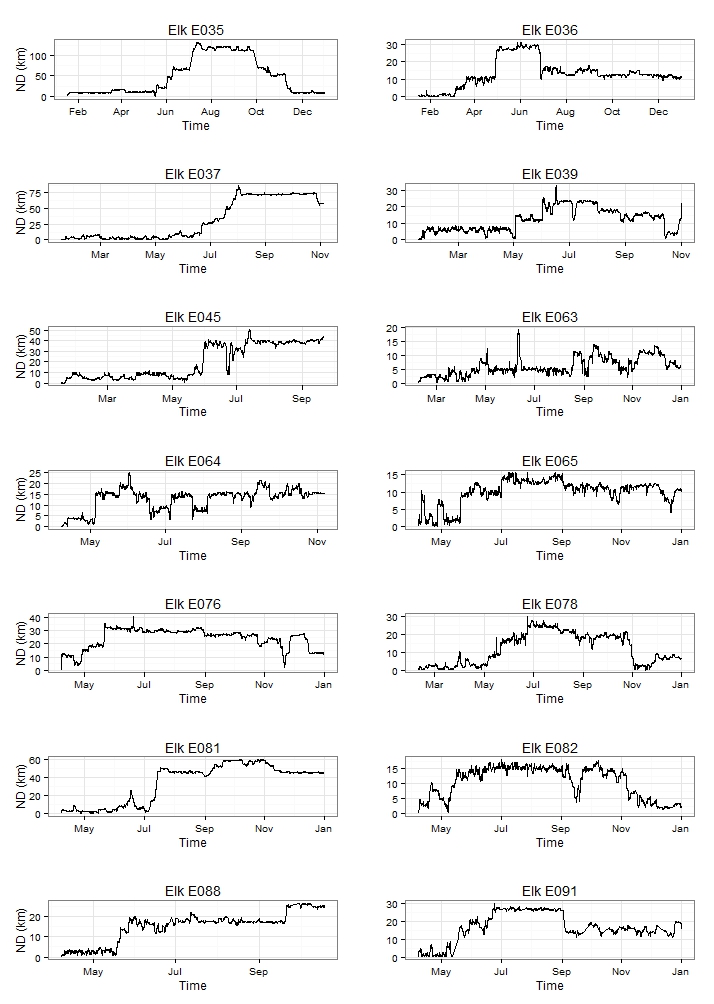


**S2 Fig (continued)**


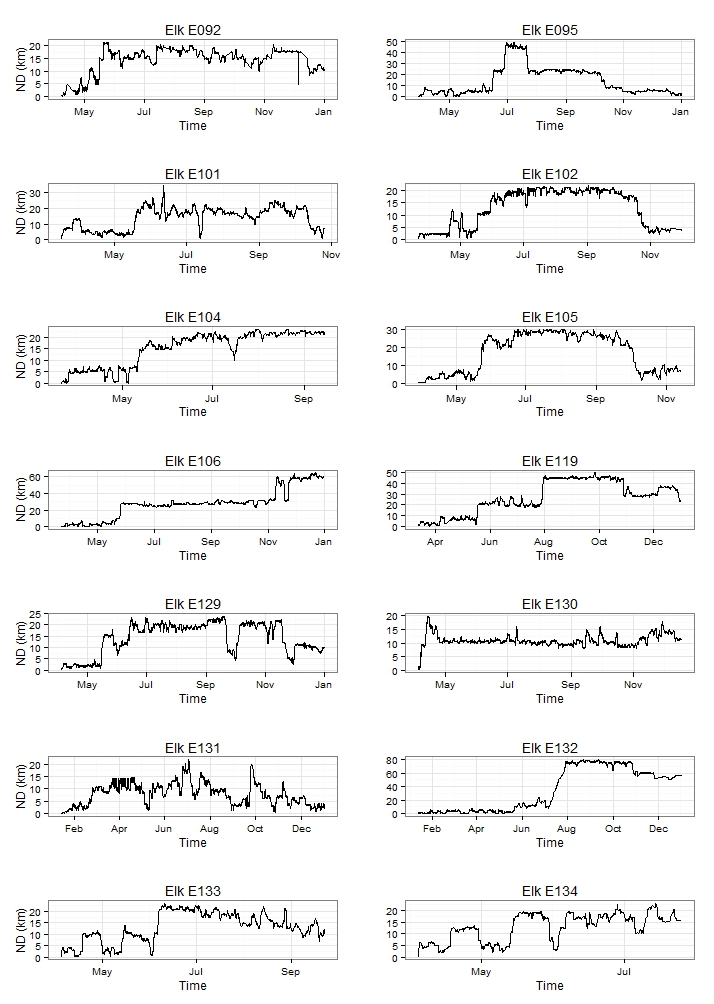


**S2 Fig (continued)**


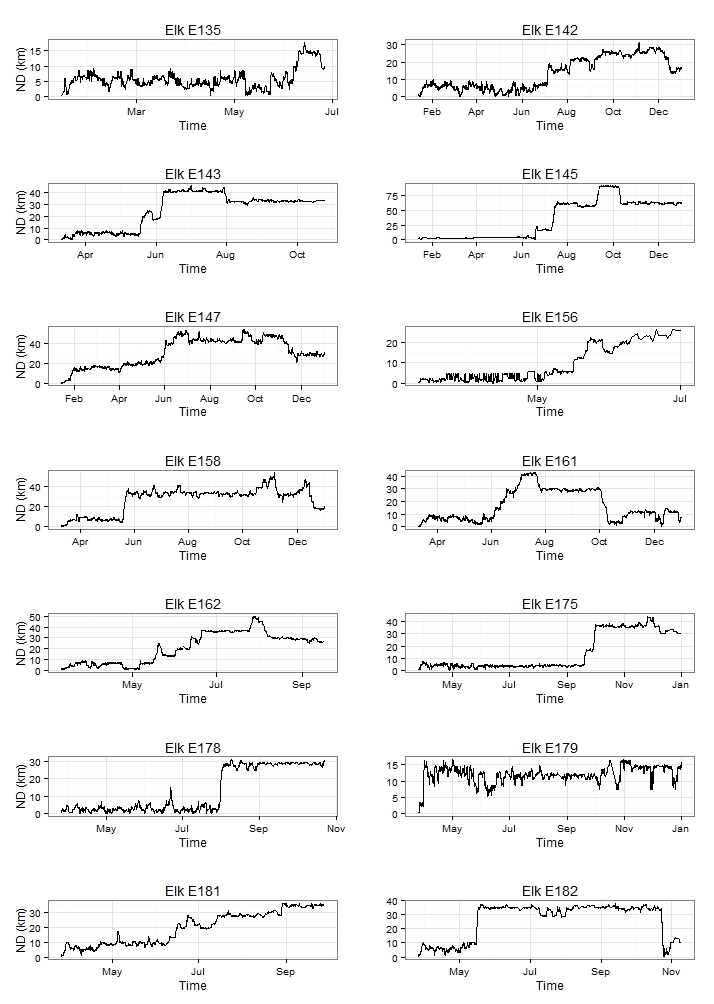


**S2 Fig (continued)**
