# Supplementary material for: Dispersal Ecology Informs Design of Large-Scale Wildlife Corridors
Source: PLoS One. 2016 Sep 22;11(9):e0162989. doi: 10.1371/journal.pone.0162989 (PMC5033395; doi:10.1371/journal.pone.0162989)
Supplement: S3 Fig — (DOCX) [file pone.0162989.s003.docx]

**
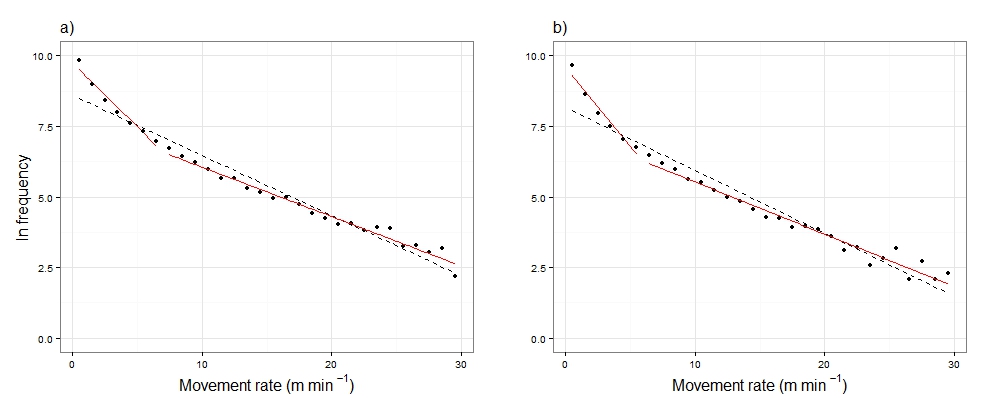
**

**S3 Fig -** Broken stick model for a) elk spring movement rates depicting the breaking point at 6.97 m min^-1^ and b) autumn movement rates with the breaking point at 5.87 m min^-1^. Elk were fitted with a satellite transmitter programmed to a 2-hour relocation schedule. The threshold movement rate (m min^-1^) identified by the broken stick model was thus converted into a distance threshold (in spring 836.4 m 2h^-1^, in autumn 704.4 m 2h^-1^) that was used to differentiate long movements (e.g., migratory, exploratory, and dispersal bouts) from short ones (e.g., foraging, resting).
