# Supplementary material for: Dispersal Ecology Informs Design of Large-Scale Wildlife Corridors
Source: PLoS One. 2016 Sep 22;11(9):e0162989. doi: 10.1371/journal.pone.0162989 (PMC5033395; doi:10.1371/journal.pone.0162989)
Supplement: S4 Fig — (DOCX) [file pone.0162989.s004.docx]

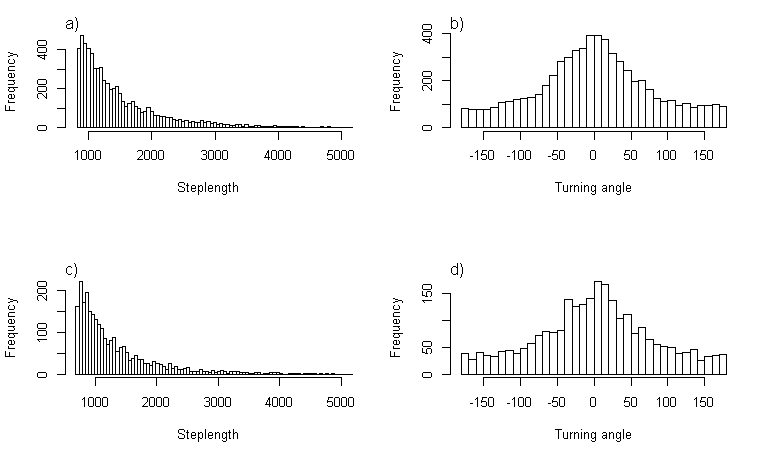


**S4 Fig -** Characteristics of steps walked by elk every 2 hours that were longer than the threshold distance identified by the broken stick model reported in S3 Fig: a) steplength and b) turning angles for spring movements; c) steplength and d) turning angles for autumn movements.
