# Supplementary material for: Dispersal Ecology Informs Design of Large-Scale Wildlife Corridors
Source: PLoS One. 2016 Sep 22;11(9):e0162989. doi: 10.1371/journal.pone.0162989 (PMC5033395; doi:10.1371/journal.pone.0162989)
Supplement: S5 Fig — (DOCX) [file pone.0162989.s005.docx]

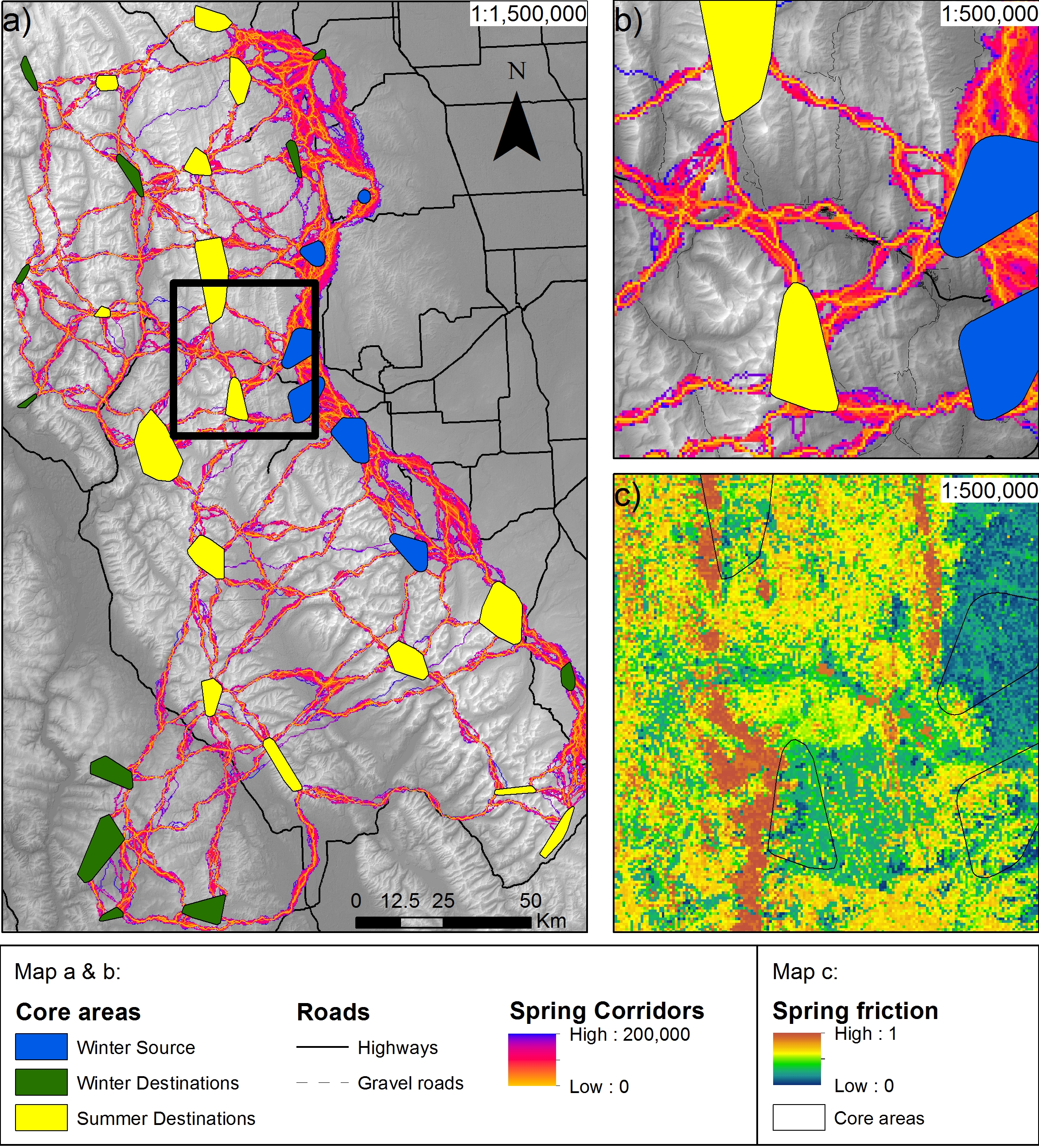


**S5 Fig -** Corridor map of elk spring movements assuming there were no roads. a) Normalized least-cost-corridors, with low values as optimal, connecting winter with summer core areas, b) close up section along highway 3, c) close up friction map produced from spring movements. Note that, compared to Fig 4, elk are predicted to move along highway 3 (map b) at the bottom valley, that would be the movement if there were no highway 3.
