# Supplementary material for: Dispersal Ecology Informs Design of Large-Scale Wildlife Corridors
Source: PLoS One. 2016 Sep 22;11(9):e0162989. doi: 10.1371/journal.pone.0162989 (PMC5033395; doi:10.1371/journal.pone.0162989)
Supplement: S1 Table — (DOCX) [file pone.0162989.s007.docx]

**S1 Table - Digital data layers.** Description of data layers used to estimate resource selection and movement behaviour in montane elk.


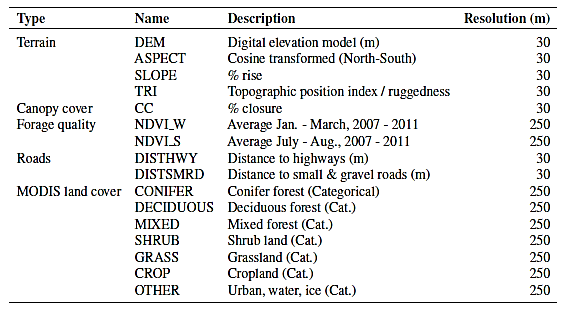


**Terrain ruggedness index (m)**

**^1^**

**^2^**

**^3^**

**^4^**

**USGS land cover**

**^5^**

***Details on digital data GIS layers.***

*1 Terrain ruggedness index TRI [sensu 1], aspect, and slope were calculated from a 30×30 m pixel spatial resolution digital elevation model (DEM) (sources - for AB: Government of Alberta; for BC: Government of Canada, Natural Resources Canada, 2006; for MT: U.S. Geological Survey, 2002)*

*2 Canopy cover (i.e., crown closure) was measured as the canopy gap fraction in each 30m pixel, and has been scaled as a continuous variable ranging from 0 to 100, in percentage. This product was generated by multiple regression of 22 Landsat Thematic Mapper 5 images and topographic derivatives from the digital elevation model DEM.*

*3 Normalized difference vegetation index (NDVI) derived from 16-day MODIS satellite imagery.*

*4 Source of road data: Governments of AB & BC: National Topographic Database 1:50,000; U.S. Census Bureau Tiger/Line files, 2000;* [*http://www.census.gov/geo/www/tiger*](http://www.census.gov/geo/www/tiger)*). HWY: double-lane highways. SMRD: single lane and gravel roads.*

*5 North American Land Change Monitoring System (re-classified and limited to 7 levels) - Source: http://landcover.usgs.gov/nalcms.php*

***References***

[1] Riley S.J., DeGloria S.D., Elliot R. 1999 A terrain ruggedness index that quantifies topographic heterogeneity. Intermountain Journal of Sciences 5, 23-27.
