# Supplementary material for: Dispersal Ecology Informs Design of Large-Scale Wildlife Corridors
Source: PLoS One. 2016 Sep 22;11(9):e0162989. doi: 10.1371/journal.pone.0162989 (PMC5033395; doi:10.1371/journal.pone.0162989)

**S2 Table -** **Resource selection functions (RSFs) during winter and summer.** Generalized linear mixed model coefficients (*β*), standard errors (SE), Wald statistics (Z) and probability values (P) comparing resources used with those sampled at random available locations across the study area from 2007 to 2011. See S1 Table for details on environmental predictors.


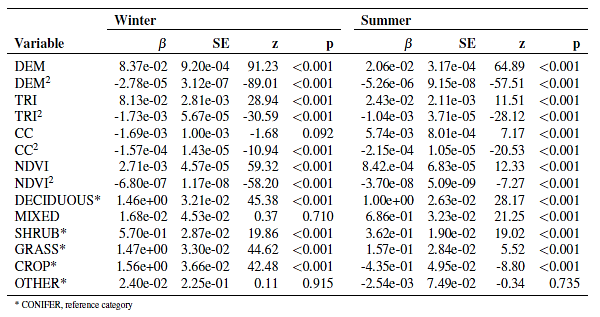

Supplement: S2 Table — (DOCX) [file pone.0162989.s008.docx]
