# Supplementary material for: Dispersal Ecology Informs Design of Large-Scale Wildlife Corridors
Source: PLoS One. 2016 Sep 22;11(9):e0162989. doi: 10.1371/journal.pone.0162989 (PMC5033395; doi:10.1371/journal.pone.0162989)

**S3 Table -**  **Step selection functions (SSFs) during spring and autumn movements.** Mixed conditional logit model coefficients (*β*), standard errors (SE), Wald statistics (Z) and probability values (P) comparing resources of walked steps by elk with those available at random steps from 2007 to 2011. See S1 Table for details on environmental predictors.


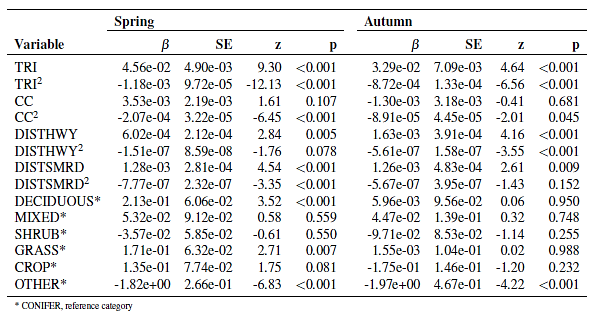

Supplement: S3 Table — (DOCX) [file pone.0162989.s009.docx]
