# Supplementary material for: Dispersal Ecology Informs Design of Large-Scale Wildlife Corridors
Source: PLoS One. 2016 Sep 22;11(9):e0162989. doi: 10.1371/journal.pone.0162989 (PMC5033395; doi:10.1371/journal.pone.0162989)

**S4 Table -** **K-fold cross-validation of step selection functions SSFs**, showing model robustness based on observed $\bar{r}_{s}$ (Spearman-rank correlations), $\bar{r}_{s}$ expected under random patterns, and 95 % confidence intervals. Validation of predictive fits is significantly achieved when 95 % CI for observed and random patterns do not overlap, which occurred in all four SSFs. Spring and autumn SSF models “with no roads” are those models where distance from roads was set to its maximum value (i.e., predicting elk movements assuming no influence of roads on behaviour). Spring and autumn SSF models “including roads” were built considering the actual distance between each step and the closest road.


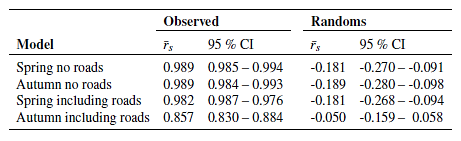

Supplement: S4 Table — (DOCX) [file pone.0162989.s010.docx]
